# Advances in artificial intelligence applications for ocular surface diseases diagnosis

**DOI:** 10.3389/fcell.2022.1107689

**Published:** 2022-12-20

**Authors:** Yuke Ji, Sha Liu, Xiangqian Hong, Yi Lu, Xingyang Wu, Kunke Li, Keran Li, Yunfang Liu

**Affiliations:** ^1^ The Laboratory of Artificial Intelligence and Bigdata in Ophthalmology, Affiliated Eye Hospital of Nanjing Medical University, Nanjing, China; ^2^ Shenzhen Eye Hospital, Jinan University, Shenzhen, China; ^3^ Department of Ophthalmology, First Affiliated Hospital of Huzhou University, Huzhou, China

**Keywords:** artificial intelligence, ocular surface disease, disease diagnosis, keratitis, keratoconus, dry eye, pterygium

## Abstract

In recent years, with the rapid development of computer technology, continual optimization of various learning algorithms and architectures, and establishment of numerous large databases, artificial intelligence (AI) has been unprecedentedly developed and applied in the field of ophthalmology. In the past, ophthalmological AI research mainly focused on posterior segment diseases, such as diabetic retinopathy, retinopathy of prematurity, age-related macular degeneration, retinal vein occlusion, and glaucoma optic neuropathy. Meanwhile, an increasing number of studies have employed AI to diagnose ocular surface diseases. In this review, we summarize the research progress of AI in the diagnosis of several ocular surface diseases, namely keratitis, keratoconus, dry eye, and pterygium. We discuss the limitations and challenges of AI in the diagnosis of ocular surface diseases, as well as prospects for the future.

## 1 Introduction

Since the beginning of the 21st century, significant changes have occurred in daily life with the rapid development of science and technology, including computer science. In 2018, the US Food and Drug Administration approved the launch of IDx-DR, which is the first ophthalmic artificial intelligence (AI) device that can automatically diagnose and grade diabetic retinopathy. Since then, there has been an upsurge in the application of AI technology in the field of ophthalmology and various research results continue to emerge. AI is a branch of computer science that mainly studies and develops new technical science to simulate and extend the theory, methods, technology, and application systems of human intelligence. Machine learning (ML), deep learning (DL), artificial neural networks, deep neural networks (DNNs), convolution neural networks (CNNs), and transfer learning all belong to this category. At present, a series of research achievements have been made in AI technology for the diagnosis and treatment of eye diseases such as diabetic retinopathy (Raman et al., 2019; Ai et al., 2021; Bhardwaj et al., 2021), retinopathy of prematurity (Redd et al., 2018; Attallah, 2021; Wang et al., 2021a), age-related macular degeneration (Burlina et al., 2018; Yan et al., 2020; Yim et al., 2020), retinal vein occlusion (Nagasato et al., 2018; Nagasato et al., 2019; Xu et al., 2022), and glaucoma (Christopher et al., 2018; Hood and De Moraes, 2018; Medeiros et al., 2021).

In general, ocular surface diseases are diseases that damage the normal structure and function of the cornea, conjunctiva, and ocular surface. In recent years, increasing studies have applied AI to assist in the diagnosis of ocular surface diseases. In this review, we summarize the application of AI in the diagnosis of four common ocular surface diseases: keratitis, keratoconus, dry eye, and pterygium. Moreover, we discuss the limitations and challenges of AI in clinical applications and future prospects. The term “diagnosis” used in this article has a broad meaning, including the designation or detection of a specific disease and other diagnostic decisions (for example, identification and screening of different disease states, subtypes, stages or degrees, and the prediction of disease progression).

**FIGURE 1:**
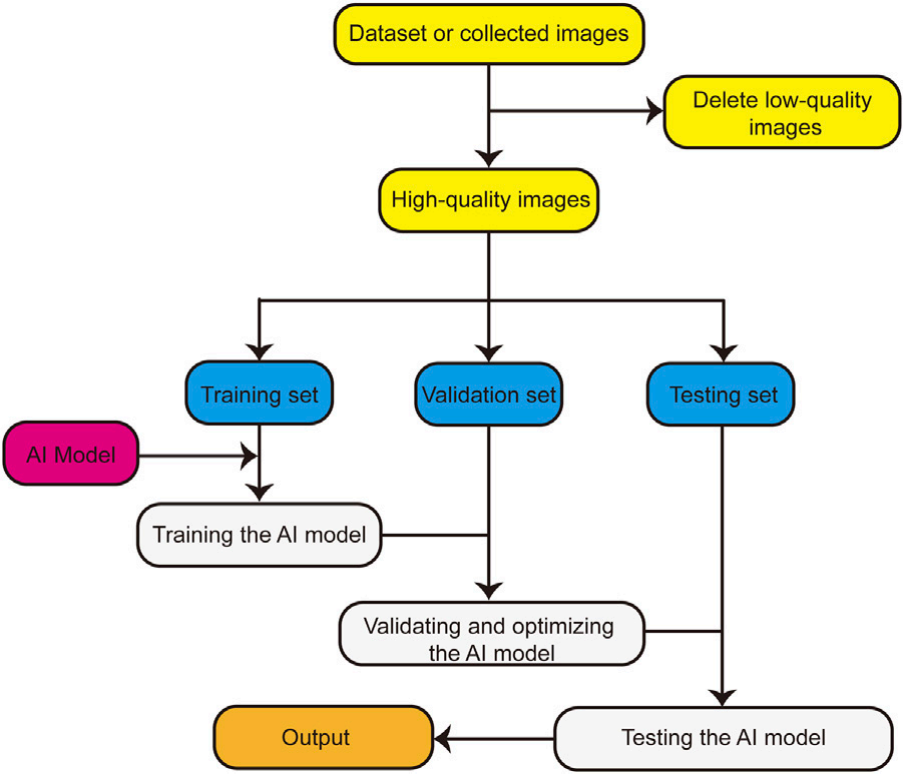
Basic flow chart of AI model.

The basic research flow of an AI model for such an application is presented in Figure 1. First, the dataset is organized, low-quality images are deleted, and the remaining high-quality images are divided into the training, verification, and testing sets. Subsequently, the AI model is trained using the training set, validated using the verification set, and optimized according to the results. Finally, the optimized AI model is tested using the testing set, and the application performance of the AI model is obtained.

**FIGURE 2:**
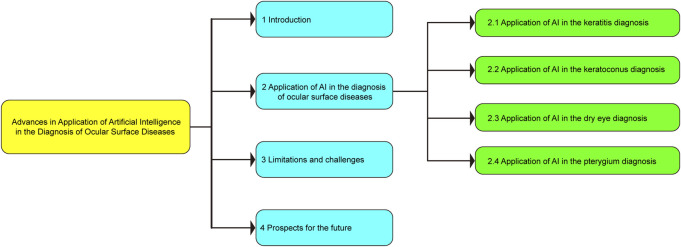
Basic framework of this review.

The basic framework of this review, which is divided into four parts, is depicted in Figure 2. The first part focuses the current status of AI and its application in ophthalmic diseases; the second part presents the research progress of AI in the diagnosis of ocular surface diseases; the third part introduces the limitations and challenges of AI in the diagnosis of ocular surface diseases; the fourth part provides an overview of the future application prospects of AI in the diagnosis of ocular surface diseases.

## 2 Application of AI in ocular surface disease diagnoses

### 2.1 Application of AI in keratitis diagnosis

Keratitis, which is the fifth most common cause of human blindness (Pascolini and Mariotti, 2012; Flaxman et al., 2017), refers to the weakening of the corneal defense ability and inflammation of the corneal tissue as a result of exogenous or endogenous pathogenic factors. The etiology of keratitis is complex; it can be caused not only by pathogenic microorganisms (such as bacteria, fungi, viruses, and chlamydia), but also by autoimmune diseases such as rheumatoid arthritis. The inflammation of adjacent tissues (such as conjunctivitis, scleritis, and iridocyclitis) may also lead to keratitis (Chidambaram et al., 2018; Khor et al., 2018). At present, the classification of keratitis has not been unified. It can be categorized as infectious, immune, malnourished, neuroparalytic, and exposed keratitis, according to its pathogenic causes. Infectious keratitis can be further subdivided into bacteria, viruses, fungi, chlamydia, and so on, according to different pathogenic microorganisms (Tena et al., 2019).

Although the etiology of keratitis is varied, the pathological processes of different types usually exhibit common characteristics. The classical pathological process can be divided into four stages: the infiltration, ulcer formation, ulcer regression, and healing stages (Li et al., 2021). The most common symptoms of keratitis in clinical manifestations include eye pain, photophobia, tears, and blepharospasm, which can persist until the inflammation subsides (Austin et al., 2017). Keratitis is often accompanied by varying degrees of vision loss. Typical signs of keratitis include ciliary hyperemia, corneal infiltration, and corneal ulcer formation. Moreover, it is often accompanied by varying degrees of vision loss. The shape and location of corneal infiltration and ulcers also differ according to the location, size, and nature of the lesion (Ting et al., 2018; Ting et al., 2021). Although keratitis exhibits typical characteristics, its diagnosis is challenging owing to its diverse clinical manifestations and atypical symptoms and signs in the early stages, and especially if the appropriate equipment is unavailable. Applying AI technology to assist in keratitis diagnosis can aid the treatment of keratitis and reduce the blindness rate (Li et al., 2021; Tahvildari et al., 2021).

Kuo et al. (Kuo et al., 2021) constructed a model for the diagnosis of bacterial keratitis based on several DL algorithms (ResNet-50, ResNeXt-50, DenseNet-121, SE-ResNet50, EfficientNet B0, EfficientNet B1, EfficientNet B2, and EfficientNet B3). They collected 1,512 slit lamp images for the training, modification, and verification of the diagnostic model. Following verification, the EfficientNet B3 model exhibited the best performance, with a sensitivity of 0.741, a specificity of 0.643, and an accuracy of 0.703. Lv et al. (Lv et al., 2020) constructed an AI model that can automatically diagnose keratitis based on the ResNet algorithm, and collected 2,088 confocal microscope images to train and test the model. Following testing, the AUC value, sensitivity, specificity, and accuracy of the model were 0.9875, 0.9186, 0.9834, and 0.9626, respectively. Kuo et al. (Kuo et al., 2020b) constructed a DL model for the diagnosis of fungal keratitis based on the DenseNet algorithm, and used 288 collected corneal images to train and test the DL model. The sensitivity, specificity, and accuracy of the diagnostic model were 0.711, 0.684, and 0.694, respectively. Liu et al. (Liu et al., 2020) proposed a DL model that can diagnose keratitis using two CNNs (AlexNet and VGGNet), and improved the diagnostic performance of the model using data enhancement and image fusion. They collected 1,213 confocal microscope images to train and validate the model. The experimental results revealed that the accuracies of the AlexNet and VGGNet models were 0.9995 and 0.9989, respectively. According to the aforementioned research, intelligent diagnosis models based on DL have exhibited good performance for keratitis diagnosis and significant application potential. Keratitis can be diagnosed as early as possible with limited medical resources, thereby reducing the occurrence of corneal blindness.

Gu et al. (Gu et al., 2020) proposed a method to distinguish infectious and non-infectious keratitis based on the Inception v3 algorithm. They collected 5,325 slit lamp images for training and testing. Following testing, the AUC values of the model for diagnosing infectious and non-infectious keratitis were 0.930 and 0.934, respectively. Hung et al. (Hung et al., 2021) constructed an AI model that can distinguish different types of keratitis using various CNNs (DenseNet-121, DenseNet-161, DenseNet-169, DenseNet-201, EfficientNet B3, Inception v3, ResNet-101, and ResNet-50). They used 1,330 slit lamp images for training and verification. The average accuracy was 0.80 and the performance of DenseNet-161 was the best, with an AUC value of 0.85. Li et al. (Li et al., 2021) presented a system using three classical DL algorithms (DenseNet-121, Inception v3, and ResNet-50) to distinguish different types of keratitis. They collected 13,557 slit lamp images for training and verification of the classification system. The DenseNet-121 model exhibited the best performance, with a sensitivity of 0.977, a specificity of 0.982, and an accuracy of 0.980. Ghosh et al. (Ghosh et al., 2022) combined three CNNs (VGG-19, ResNet-50, and DenseNet-121) to create an AI model that can distinguish bacterial keratitis from fungal keratitis. They used 2,167 slit lamp images for training and testing. The results demonstrated that the model sensitivity was 0.77, the F1 score was 0.83, and the AUC value was 0.904. The above AI model exhibits good performance in the classification of keratitis, which is close to that of clinical practice, and is expected to become a powerful auxiliary tool in clinical work.

Xu et al. (Xu et al., 2021a) developed an AI model that can automatically detect and evaluate corneal inflammatory cells in patients with keratitis using five DL algorithms (VGG-16, ResNet-101, Inception v3, Xception, and Inception-ResNet v2). They used 4,011 confocal microscope images to train and verify the model. The Inception-ResNet v2 model exhibited the best performance, with an AUC value of 0.9646, an accuracy of 0.9767, a sensitivity of 0.9174, and a specificity of 0.9931. Tiwari et al. (Tiwari et al., 2022) constructed an AI model based on a CNN that can distinguish active keratitis from scar healing. They collected 2,445 corneal images for the model training and verification. Following verification, the F1 score of the model was 0.843, the sensitivity was 0.935, the specificity was 0.8442, and the AUC value was 0.9731. The above results suggest that AI technology also offers good application potential in evaluating the activity of keratitis. The above studies are summarized in Table 1.

**TABLE 1:** Summary of application of AI models in keratitis.

Authors	Task	Sample size	AI algorithms	Diagnostic performance
Kuo et al. (2021)	Diagnosis	1,512 images	ResNet-50, ResNeXt-50, DenseNet-121, SE-ResNet-50, EfficientNet B0, EfficientNet B1, EfficientNet B2, EfficientNet B3	Sensitivity = 0.741, Specificity = 0.643, Accuracy = 0.703
Lv et al. (2020)	Diagnosis	2,088 images	ResNet	AUC = 0.9875 Sensitivity = 0.9186 Specificity = 0.9834 Accuracy = 0.9626
Kuo et al. (2020b)	Diagnosis	288 images	DenseNet	Sensitivity = 0.711 Specificity = 0.684 Accuracy = 0.694
Liu et al. (2020)	Diagnosis	1,213 images	AlexNet	Accuracy of AlexNet = 0.9995
			VGGNet	Accuracy of VGGNet = 0.9989
Gu et al. (2020)	Classification	5,325 images	Inception v3	AUC of infectious keratitis = 0.930
				AUC of non-infectious keratitis = 0.934
Hung et al. (2021)	Classification	1,330 images	DenseNet-121, DenseNet-161, DenseNet-169, DenseNet-201, EfficientNet B3, Inception v3, ResNet-101, ResNet-50	Average accuracy = 0.80, AUC of DenseNet-161 = 0.85
Li et al. (2021)	Classification	13,557 images	DenseNet-121, Inception v3, ResNet-50	Sensitivity = 0.977 Specificity = 0.982 Accuracy = 0.980
Ghosh et al. (2022)	Classification	2,167 images	VGG19, ResNet-50, DenseNet-121	Sensitivity = 0.77, F1 score = 0.83, AUC = 0.904
Xu et al. (2021a)	Detection	4,011 images	VGG-16,ResNet-101, Inception v3, Xception, Inception-ResNet v2	AUC = 0.9646, Accuracy = 0.9767, Sensitivity = 0.9174, Specificity = 0.9931
Tiwari et al. (2022)	Classification	2,445 images	CNNs	F1 score = 0.843, Sensitivity = 0.935, Specificity = 0.8442, AUC = 0.9731

### 2.2 Application of AI in keratoconus diagnosis

Keratoconus is a congenital developmental disorder that is characterized by localized conical protuberances with thinning of the corneal stroma in the protuberant area. Conus protuberances may lead to severe irregular astigmatism and high myopia, thereby resulting in severe vision loss (Pinero et al., 2012; Hashemi et al., 2020). The disease generally occurs before and after puberty and occurs in both eyes, with a progressive decline in visual acuity (Chatzis and Hafezi, 2012). It can be corrected by myopic lenses in the early stages and contact lenses need to be worn owing to irregular astigmatism in the later stages (Papali’i-Curtin et al., 2019). The typical characteristics of the disease are central or paracentric conic dilatation, whereby the cone may be large or small, round or oval, and the thinning area of the corneal stroma is most obvious at the top of the cone. Patients with advanced keratoconus can see Munson’s sign, Vogt’s striae, or Fleischer’s ring and other clinical signs, which can aid in diagnosing keratoconus (de Sanctis et al., 2008; Gordon-Shaag et al., 2012; Chan et al., 2021). Although clinical diagnosis is straightforward for obvious keratoconus, it is difficult to diagnose atypical early keratoconus. At present, the most effective method for the early diagnosis of the disease is corneal topography, which reveals that the central corneal topography is distorted and the lower quadrant becomes steep. The corneal steepness expands to the subnasal, superior temporal, and superior nasal quadrants with the progression of the disease. Other examination methods include keratometers, retinography, and Placido discs (Brunner et al., 2018; Mohammadpour et al., 2018; Rocha-de-Lossada et al., 2021). Patients with early keratoconus can wear frame glasses or keratoplasty lenses according to the optometry results to improve their visual acuity (Goh et al., 2020). Moreover, intracorneal ring implants and corneal cross-linking or other methods can be used to delay the progress of the disease (Ferdi et al., 2019). If patients with early keratoconus do not receive effective intervention, the late stage will lead to severe vision loss, requiring keratoplasty, or even blindness. Therefore, the early screening, detection, and effective intervention of keratoconus are particularly important.

Tan et al. (Tan et al., 2022) proposed a diagnostic model for keratoconus based on the 5-FNN neural network model. They collected corneal videos of 354 eyes for the model training and testing. The results revealed that the diagnostic accuracy, sensitivity, and specificity of the model were 0.996, 0.993, and 1.000, respectively. Kamiya et al. (Kamiya et al., 2019) developed a diagnostic classification model based on ResNet-18 to assist in the diagnosis and classification of keratoconus. They collected 543 anterior segment optical coherence tomography (As-OCT) images for the model training and testing. According to the results, the diagnostic accuracy of the model was 0.991 and the classification accuracy was 0.874. Dos Santos et al. (Dos Santos et al., 2019) designed an AI model that can diagnose keratoconus based on U-Net. They collected and marked 20,160 images for the model training and testing. Following testing, the accuracy of the model was 0.9956. The high accuracy and excellent performance of the above AI models demonstrate that AI technology can be used extensively in the clinical diagnosis and treatment of keratoconus, thereby greatly reducing the work stress of clinicians.

As early keratoconus often exhibits no typical symptoms and signs, screening to distinguish patients with keratoconus will help them to receive earlier treatment. Kuo et al. (Kuo et al., 2020a) constructed an AI model that can screen keratoconus based on three CNNs (VGG-16, Inception v3, and ResNet-152), and collected 354 corneal topographic maps for model training and external testing. The results revealed that the ResNet-152 model achieved the best performance, with an accuracy of 0.958, a sensitivity of 0.944, a specificity of 0.972, and an AUC value of 0.995. Chen et al. (Chen et al., 2021) presented a model that can detect coning modeling using CNNs. The model was trained and tested using the whole Liverpool (United Kingdom) and New Zealand (NZ) datasets. The results demonstrated that the model accuracy was 0.9785. Lavric et al. (Lavric and Valentin, 2019) constructed a screening model that can rapidly screen keratoconus based on CNNs, and collected 4,350 corneal topographic maps to train and test the model. The results indicated that the model accuracy was 0.9933. Al-Timemy et al. (Al-Timemy et al., 2021) developed a detection model that can recognize keratoconus based on the EfficientNet B0 DL algorithm. They collected 4,844 corneal topography maps for the training, debugging, and verification of the model. The AUC value, F1 score, and accuracy of the model were 0.99, 0.99, and 0.985, respectively. Abdelmotaal et al. (Abdelmotaal et al., 2020) constructed an AI model that can recognize keratoconus based on CNNs, and used 19,310 corneal topographic maps for training and testing. The test results demonstrated that the model accuracy was 0.958. In view of the good results of the above AI models in keratoconus identification and screening, timely diagnosis and treatment is possible.

Castro-Luna et al. (Castro-Luna et al., 2021) developed a model that can classify subclinical keratoconus using the random forest (RF) model. They collected clinical data of 81 eyes to train and verify the model. Kamiya et al. (Kamiya et al., 2021) presented a neural network prediction model to predict the progression of keratoconus, and collected 218 As-OCT images for training and verification. The results revealed that the prediction accuracy of the model was 0.794. Kato et al. (Kato et al., 2021) constructed an AI model that can predict the progression of keratoconus based on the VGG-16 neural network model, and collected 274 corneal tomography images for training and verification. According to the results, the AUC value, sensitivity, and specificity of the model were 0.814, 0.778, and 0.696, respectively. Yousefi et al. (Yousefi et al., 2018) developed an AI model using ML to predict the severity of keratoconus. They collected and processed 3,156 corneal topographic maps for the model training and verification. The specificity and sensitivity of the model were 0.941 and 0.977, respectively. Herber et al. (Herber et al., 2021) presented an AI model that can predict the severity of keratoconus through two types of ML (linear discriminant analysis (LDA) and RF algorithms), and collected clinical data of 434 eyes for training and verification. Following verification, the accuracies of the LDA and RF models were 0.71 and 0.78, respectively. The above studies demonstrate that AI models can achieve satisfactory results in the classification and prediction of the progression of keratoconus. Thus, such models can be used to create effective treatment plans for keratoconus patients. The above studies are summarized in Table 2.

**TABLE 2:** Summary of application of AI models in keratoconus.

Authors	Task	Sample size	AI algorithms	Diagnostic performance
Tan et al. (2022)	Diagnosis	354 eyes	5-FNN	Accuracy = 0.996, Sensitivity = 0.993, Specificity = 1.000
Kamiya et al. (2019)	Diagnosis	543 images	ResNet-18	Accuracy = 0.991
				Accuracy = 0.874
Dos Santos et al. (2019)	Diagnosis	20,160 images	U-Net	Accuracy = 0.9956
Kuo et al. (2020a)	Detection	354 maps	VGG-16, Inception v3, ResNet-152	Accuracy = 0.958, Sensitivity = 0.944
				Specificity = 0.972
				AUC = 0.995
Chen et al. (2021)	Detection	Liverpool and New Zealand datasets	CNNs	Accuracy = 0.9785
Lavric and Valentin, (2019)	Detection	4,350 maps	CNNs	Accuracy = 0.9933
Al-Timemy et al. (2021)	Detection	4,844 maps	EfficientNet B0	AUC = 0.99
				F1 score = 0.99
				Accuracy = 0.985
Abdelmotaal et al. (2020)	Detection	19,310 maps	CNNs	Accuracy = 0.958
Castro-Luna et al. (2021)	Classification	81 eyes	RF	Accuracy = 0.89
Kamiya et al. (2021)	Prediction	218 images	Neural network	Accuracy = 0.794
Kato et al. (2021)	Prediction	274 images	VGG-16	AUC = 0.814
				Sensitivity = 0.778, Specificity = 0.696
Yousefi et al. (2018)	Prediction	3,156 maps	ML	Specificity = 0.941
				Sensitivity = 0.977
Herber et al. (2021)	Prediction	434 eyes	LDA, RF	Accuracy of LDA = 0.71
				Accuracy of RF = 0.78

### 2.3 Application of AI in the diagnosis of dry eye

Dry eye, which is also known as keratoconjunctivitis sicca, refers to the decline in tear film stability caused by an abnormal quality and quantity of tears or abnormal dynamics resulting from any cause. It is accompanied by eye discomfort, resulting in ocular surface tissue lesions of various diseases (Craig et al., 2017a; Craig et al., 2017b). Dry eye disease is caused by many complex pathological processes. It can be roughly divided into abnormal tear dynamics and an abnormal ocular surface epithelium (Hu et al., 2021), both of which often play a role overall. Recent studies have demonstrated that changes in the eye surface, immune-based inflammatory response, apoptosis, decreased levels of sex hormones, and meibomian gland dysfunction are the main causes of xerophthalmia (Cardona et al., 2011; Argiles et al., 2015; Rodriguez et al., 2018; DeAngelis et al., 2019). However, the relationship or causal relationship between the factors is not yet fully understood. At present, no consensus exists on the diagnostic classification criteria of dry eye. According to the etiology, dry eye is mainly divided into water sample deficiency dry eye, mucin deficiency dry eye, lipid deficiency dry eye, and dry eye caused by abnormal tear dynamics. The most common symptoms of dry eye are eye fatigue, foreign body sensation, dryness, burning, eye distension, eye pain, photophobia, and eye redness (Tepelus et al., 2017). Dry eyes slightly affect visual acuity in the early stage. Filamentous keratitis may occur after the development of the disease. Corneal ulcers, corneal thinning, perforation, and occasional secondary bacterial infection may occur in the late stage, and visual acuity will be seriously affected after the formation of corneal scar, thereby resulting in a decline in the quality of life of patients (Nichols et al., 2011; Stapleton et al., 2017). The main clinical examination methods for dry eye include the tear secretion test, tear film rupture time, tear river height measurement, Schirmer test, tear osmotic pressure, and fluorescein staining (Nichols et al., 2004; Sullivan et al., 2010; Zeev et al., 2014; Vehof et al., 2020). Doctors need to spend more time and energy on examination and analysis in the clinical diagnosis of dry eye. Numerous research data have shown that dry eye has a high incidence and consumes substantial manpower and financial resources every year; thus, it is necessary to improve the diagnosis and treatment efficiency of dry eye.

AI has been increasingly applied to dry eye with remarkable effects. Chase et al. (Chase et al., 2021) constructed a DL model for the diagnosis of dry eye. They collected 27180 As-OCT images for the model training and testing. The results demonstrated that the accuracy, sensitivity, and specificity of the model in the diagnosis of dry eye were 0.8462, 0.8636, and 0.8235, respectively. Zhang et al. (Zhang et al., 2022) established a dry eye diagnosis model using a U-Net image segmentation algorithm and ResNet image classification algorithm. The models were trained and evaluated using blinking videos of 357 patients with dry eye and 152 normal persons, and the accuracies were 0.963 and 0.960, respectively. Da Cruz et al. (da Cruz et al., 2020a) used six DL models (the support vector machine (SVM), RF, naive Bayes, multilayer perceptron, random tree, and radial basis function network) for the classification of tear film images to assist in the diagnosis of dry eye. They used the VOPTICAL_GCU database for training and verification. The RF model achieved the best classification effect, with an accuracy of 0.990, an AUC value of 0.999, a kappa value of 0.995, and an F-measure of 0.996. Da Cruz et al. (da Cruz et al., 2020b) also used the six DL models to classify tear film lipid layers automatically for the diagnosis of dry eye. They trained and tested various DL models on the VOPTICAL_GCU datasets. The results revealed that the classification effect of the RF model was the best, with an accuracy of 0.97 and an AUC value of 0.99. Based on the above research, AI models exhibit high accuracy and superior performance in the diagnosis of dry eye, and can be used in the clinical diagnosis and treatment of dry eye in the future.

Koprowski et al. (Koprowski et al., 2016) developed a method for the automatic quantitative assessment of meibomian gland dysfunction (MGD) based on DL, and used 172 images (upper and lower eyelid images of 86 participants) for training and verification. The results revealed that the sensitivity of this method was 0.993 and the specificity was 0.975, which was faster and more accurate than an ophthalmologist. Wang et al. (Wang et al., 2019) proposed a method that can accurately evaluate meibomian gland atrophy based on a DNN. They collected 706 upper eyelid images for the model training, adjustment, and verification. The results demonstrated that the segmentation accuracy of the meibomian gland atrophy was 0.954 and the overall grading accuracy was 0.956. Waruoka et al. (Maruoka et al., 2020) constructed various DL models to detect obstructive MGD. Following training and verification using 137 images, the performance of DenseNet-201 was the best, with an AUC value of 0.966, a sensitivity of 0.942, and a specificity of 0.821. Setu et al. (Setu et al., 2021) constructed an algorithm for meibomian gland segmentation based on DL. A total of 728 clinical images were used to train and evaluate the model. According to the results, the average precision, recall, and F1 score were 0.83, 0.81, and 0.84, respectively. The function of the meibomian gland is closely related to the incidence of dry eye. These studies, which are summarized in Table 3, demonstrate that AI technology can be used to effectively evaluate the function of the meibomian gland, reduce the analysis time, and improve the diagnostic accuracy of doctors.

**TABLE 3:** Summary of application of AI models in dry eye.

Authors	Task	Sample size	AI algorithms	Diagnostic performance
Chase et al. (2021)	Diagnosis	27,180 images	DL	Accuracy = 0.8642, Sensitivity = 0.8636, Specificity = 0.8235
Zhang et al. (2022)	Diagnosis	507 videos	U-Net	Accuracy of U-Net = 0.963
			ResNet	Accuracy of ResNet = 0.960
da Cruz et al. (2020a)	Diagnosis	VOPTICAL_GCU database	SVM, RF, naive Bayes, multilayer perceptron, random tree, radial basis function network	Accuracy = 0.990
				AUC = 0.999
				Kappa = 0.995
				F-measure = 0.996
da Cruz et al. (2020b)	Diagnosis	VOPTICAL_GCU database	SVM, RF, naive Bayes, multilayer perceptron, random tree, radial basis function network	Accuracy = 0.97
				AUC = 0.99
Koprowski et al. (2016)	Assessment	172 images	DL	Sensitivity = 0.993
				Specificity = 0.975
Wang et al. (2019)	Assessment	706 images	DNNs	Accuracy of meibomian gland atrophy segmentation = 0.954
				Overall grading accuracy = 0.956
Maruoka et al. (2020)	Detection	137 images	DL	AUC = 0.966
				Sensitivity = 0.942
				Specificity = 0.821
Setu et al. (2021)	Detection	728 images	DL	Accuracy = 0.83
				Recall = 0.81
				F1 score = 0.84

### 2.4 Application of AI in pterygium diagnosis

Pterygium is a chronic inflammatory disease named for its insect wing shape. It is mainly characterized by fibrovascular hyperplasia of conjunctival tissue and the invasion of the surrounding corneal tissue, which is also known as proliferative disease (Yue and Gao, 2019; Wang et al., 2021b). Pterygium usually consists of three parts: the head, neck, and body, which often invade the cornea and limbus cornea (Seet et al., 2012). Its incidence is closely related to the geographical latitude, especially near the equator between 30 and 35 degrees. Furthermore, the disease is more common in outdoor working people (such as fishermen and farmers) (Coroneo, 2011; Delic et al., 2017). However, the specific cause of the disease remains unknown and it may be related to ultraviolet exposure, smoke, viral infections, ocular degeneration, sex, and age (Sjo et al., 2007; Huang et al., 2013; Rezvan et al., 2018). Clinically, the disease occurs in both eyes, especially on the nasal side. In the early stage, there are generally no obvious symptoms or only a slight foreign body sensation. When the lesion invades the corneal pupil area, corneal astigmatism or direct occlusion of the pupil area will occur, thereby resulting in a decline in visual acuity (Kampitak et al., 2016). Pterygium can divided into the progressive and static types according to the development of abnormal tissue (Safi et al., 2016). Progressive pterygium exhibits protuberance of the head and infiltration at the front, Stocker lines at times, and hyperemia and hypertrophy of the body, with gradual growth into the cornea. Static pterygium exhibits a flat head, thin body, and static non-development (Mohd Radzi et al., 2019). At present, the clinical diagnosis of pterygium is mainly dependent on anterior segment photography (Abdani et al., 2022). Surgery is the main treatment for the disease. Small and static pterygium generally do not require treatment, but sand, sunlight, and other stimulation should be reduced as far as possible. Furthermore, when the pterygium invades the pupil area, it should be resected in time (Graue-Hernandez et al., 2019). However, surgical resection may still result in postoperative complications in patients with advanced pterygium, such as a high recurrence rate, corneal scarring, and astigmatism (Hirst, 2003; Mahar and Manzar, 2013; Resnikoff et al., 2020). Therefore, it is very important to screen pterygium and evaluate the timing of surgery in the early stage.

In recent years, with the rapid development of AI, it has been increasingly applied to assist in the clinical screening, diagnosis, and prognosis of pterygium. Zheng et al. (Zheng et al., 2021) constructed two diagnostic models (MobileNet 1 and MobileNet 2) that can aid in the diagnosis of pterygium. They collected 436 images of the anterior segment of the eyes for the testing and training of the diagnostic models. The MobileNet 2 model achieved the best performance, with a sensitivity of 0.8370, a specificity of 0.9048, and an F1 score of 0.8250. Wan et al. (Wan et al., 2022) constructed a diagnosis system for pterygium using U-Net, which was employed to assist doctors in creating surgical treatment strategies for pterygium patients. They collected 489 anterior segment images to test and verify the diagnosis system. The experimental results revealed that the Dice coefficients of the pterygium and corneal segmentation were 0.9020 and 0.9620, respectively, and the kappa consistency coefficient between the diagnosis results of the system and those of doctors was 0.918, which indicates that the system offers practical application significance. Xu et al. (Xu et al., 2021b) studied a diagnostic system that can intelligently diagnose pterygium using a DL algorithm. They collected 1,220 anterior segment images for the system training and testing. Compared with the expert diagnosis results, the diagnostic accuracy of the system was 0.9468 and the specificity was high. The above research demonstrates that AI technology can be used as an auxiliary diagnostic tool to assist clinicians with diagnosing pterygium, thereby significantly reducing their work stress and improving their efficiency.

Zaki et al. (Wan Zaki et al., 2018) built a system for pterygium screening based on a DL algorithm, and evaluated the system using a using an SVM and an artificial neural network. They used the UBIRIS, MILES, and Brazil Pterygium databases to train, modify and test the system. The results demonstrated that the accuracy, sensitivity, specificity, and AUC value of the system were 0.9127, 0.887, 0.883, and 0.956, respectively. Abdani et al. (Abdani et al., 2021) developed a system that can automatically screen pterygium through the DL algorithm, and used 328 images of the anterior segment of the eye for training and verification. The accuracy of the system was 0.9330. Fang et al. (Fang et al., 2021) created a pterygium detection model based on DL, and collected 9443 images of the anterior segment of the eye for the model training and testing. The AUC value, sensitivity, and specificity of the model were 0.995, 0.985, and 0.990, respectively. These studies demonstrate that AI models have exhibited good performance in pterygium screening. It is expected that such approaches can be used in pterygium screening in areas where medical resources are scarce or the economy is challenged to achieve early diagnosis and timely medical treatment for pterygium patients.

Jais et al. (Jais et al., 2021) developed a model that can predict the best corrected visual acuity of patients with pterygium using four different ML algorithms (the decision tree, SVM, logistic regression, and naive Bayes). They used the data of 93 patients with different types of pterygium as the dataset for the model. The final results showed that the SVM model achieved the best performance, with an accuracy of 94.44% ± 5.86%, a specificity of 100%, and a sensitivity of 92.14% ± 8.33%. Hung et al. (Hung et al., 2022) developed a DL system for grading pterygium and predicting postoperative recurrence. The system used 237 images for training and testing. According to the results, the sensitivity, F1 score, and accuracy for the pterygium grading were 0.8000–0.9167, 0.8182 to 0.9434, and 0.8667 to 0.9167, respectively, whereas the sensitivity and specificity for predicting the postoperative recurrence of pterygium were 0.6667 and 0.8182, respectively. Thus, AI models can aid in predicting the recurrence and prognosis of pterygium, and can help clinicians to deal with various postoperative complications better, so as to create the most effective treatment plan. The above studies are summarized in Table 4.

**TABLE 4:** Summary of application of AI models in pterygium.

Authors	Task	Sample size	AI algorithms	Diagnostic performance
Zheng et al. (2021)	Diagnosis	436 images	MobileNet 1, MobileNet 2	Sensitivity = 0.8370, Specificity = 0.9048
				F1 score = 0.8254
				AUC = 0.8720
Wan et al. (2022)	Diagnosis	489 images	U-Net	Dice of pterygium = 0.9020
				Dice of cornea = 0.9620, Kappa = 0.918
Xu et al. (2021b)	Diagnosis	1,220 images	DL	Accuracy = 0.9468
Wan Zaki et al. (2018)	Detection	UBIRIS, MILES, and Brazil Pterygium databases	SVM, neural network	Accuracy = 0.9127, Sensitivity = 0.887, Specificity = 0.883
				AUC = 0.956
Abdani et al. (2021)	Detection	328 images	DL	Accuracy = 0.9330
Fang et al. (2021)	Detection	9,443 images	DL	AUC = 0.995
				Sensitivity = 0.985, Specificity = 0.990
Jais et al. (2021)	Prognosis and recurrence	93 patients	Decision tree, SVM, logistic regression, naive Bayes	Accuracy = 94.44% ± 5.86%
				Specificity = 100%
				Sensitivity = 92.14% ± 8.33%
Hung et al. (2022)	Prognosis and recurrence	237 images	DL	Sensitivity = 0.6667
				Specificity = 0.8182

## 3 Limitations and challenges

According to the aforementioned diverse applications of AI in ocular surface disease diagnoses, AI has shown considerable advantages for ocular surface and other ophthalmic disease diagnoses, especially through data and image analysis. However, although many studies on the application of AI to the diagnosis of ocular surface diseases have exhibited satisfactory results, they still have numerous limitations and challenges. 1) Datasets suffer from image quality problems (Ghosh et al., 2022; Dong et al., 2022). Some of the images in the training, verification, and test sets used in some AI studies suffered from quality problems, such as unclear or incomplete images, which significantly impacted the research results. 2) The external verification of algorithms face many challenges (Tan et al., 2022; Martins et al., 2022). The DL algorithms in several studies was verified and tested on open datasets. When they are applied to actual clinical diagnosis and treatment, their performance will be reduced owing to the differences in image quality, shooting equipment, patient cooperation etc. 3) The sample size used in some studies was small (Zhang et al., 2022; Kang et al., 2022). The datasets used in some studies contained small sample sizes, resulting in unstable performance of the AI models and large differences in results. 4) Heterogeneity of patients (Wan et al., 2022; Sheng et al., 2022). Every person is different, and most individuals have considerable differences among each other. This human heterogeneity is likely to result in a decline in the accuracy of AI model verification and testing for clinical diagnosis and treatment. 5) Biases exist in AI model datasets (Hung et al., 2022; Keel et al., 2018; Pur et al., 2022). The AI models are most likely to be successful when they are trained and validated using high-quality datasets. However, many studies used small or common datasets (wherein some data may be biased), which caused certain biases in their results, resulting in low external applicability of AI models.

## 4 Prospects for the future

Although the application of AI to the clinical diagnosis of ophthalmic diseases, such as ocular surface diseases, still faces numerous challenges. The current AI studies on ocular surface disease diagnoses indicate that AI can obtain the disease characteristics from the training set and apply them to the verification or testing set to diagnose the corresponding disease. AI can classify images into different types according to the disease characteristics, such as disease classification and stage. Additionally, AI can also detect and segment the anatomical structure in the image, such lesion shape, to realize the automatic quantization of image biomarkers and perform auxiliary diagnosis. Therefore, based on these advantages, the application of AI technology in clinical diagnosis and treatment offers infinite potential and significant prospects. With the continual progress of science and technology, the ongoing improvements in AI, and the establishment and improvement of relevant legal systems, AI will be better applied to the clinical diagnosis and treatment of ophthalmology, especially in economically challenged areas and those that lack medical resources, in the near future. The application of AI will greatly improve the level of diagnosis and treatment in such areas, thereby aiding more patients to detect diseases as soon as possible, which is essential for early diagnosis and treatment. Moreover, if clinical diagnosis and treatment course can be entirely established through AI, the work stress of clinical medical staff will be significantly reduced and their work efficiency will improve, allowing them to perform the best diagnosis and offer the best treatment plan for patients.

AI offers the potential to improve the diagnosis level of ophthalmic diseases significantly. In the future, with the expansion of AI in the field of ophthalmology, in addition to image processing technology, other AI technologies will be researched and applied in the field of ophthalmology. The full application of AI will result in fundamental changes in the clinical ophthalmology diagnosis and treatment.

## References

[B1] Abdani S. R. Zulkifley M. A. Shahrimin M. I. Zulkifley N. H. (2022). Computer-assisted pterygium screening system: A review. Diagn. (Basel) 12, 639. 10.3390/diagnostics12030639 PMC8947201 35328192

[B2] Abdani S. R. Zulkifley M. A. Zulkifley N. H. (2021)., 11. Basel), 1104. 10.3390/diagnostics11061104 Group and shuffle convolutional neural networks with pyramid pooling module for automated pterygium segmentation Diagnostics 34204479 PMC8235574

[B3] Abdelmotaal H. Mostafa M. M. Mostafa A. N. R. Mohamed A. A. Abdelazeem K. (2020). Classification of color-coded scheimpflug camera corneal tomography images using deep learning. Transl. Vis. Sci. Technol. 9, 30. 10.1167/tvst.9.13.30 PMC7757611 33384884

[B4] Ai Z. Huang X. Fan Y. Feng J. Zeng F. Lu Y. (2021). DR-IIXRN : Detection algorithm of diabetic retinopathy based on deep ensemble learning and attention mechanism. Front. Neuroinform. 15, 778552. 10.3389/fninf.2021.778552 35002666 PMC8740273

[B5] AL-Timemy A. H. Mosa Z. M. Alyasseri Z. Lavric A. Lui M. M. Hazarbassanov R. M. (2021). A hybrid deep learning construct for detecting keratoconus from corneal maps. Transl. Vis. Sci. Technol. 10, 16. 10.1167/tvst.10.14.16 PMC8684312 34913952

[B6] Argiles M. Cardona G. Perez-Cabre E. Rodriguez M. (2015). Blink rate and incomplete blinks in six different controlled hard-copy and electronic reading conditions. Invest. Ophthalmol. Vis. Sci. 56, 6679–6685. 10.1167/iovs.15-16967 26517404

[B7] Attallah O. (2021). Diarop: Automated deep learning-based diagnostic tool for retinopathy of prematurity. Diagnostics 11, 2034. 10.3390/diagnostics11112034 34829380 PMC8620568

[B8] Austin A. Lietman T. Rose-Nussbaumer J. (2017). Update on the management of infectious keratitis. Ophthalmology 124, 1678–1689. 10.1016/j.ophtha.2017.05.012 28942073 PMC5710829

[B9] Bhardwaj C. Jain S. Sood M. (2021). Deep learning-based diabetic retinopathy severity grading system employing quadrant ensemble model. J. Digit. Imaging 34, 440–457. 10.1007/s10278-021-00418-5 33686525 PMC8289963

[B10] Brunner M. Czanner G. Vinciguerra R. Romano V. Ahmad S. Batterbury M. (2018). Improving precision for detecting change in the shape of the cornea in patients with keratoconus. Sci. Rep. 8, 12345. 10.1038/s41598-018-30173-7 30120293 PMC6097997

[B11] Burlina P. Joshi N. Pacheco K. D. Freund D. E. Kong J. Bressler N. M. (2018). Utility of deep learning methods for referability classification of age-related macular degeneration. JAMA Ophthalmol. 136, 1305–1307. 10.1001/jamaophthalmol.2018.3799 30193354 PMC6248178

[B12] Cardona G. Garcia C. Seres C. Vilaseca M. Gispets J. (2011). Blink rate, blink amplitude, and tear film integrity during dynamic visual display terminal tasks. Curr. Eye Res. 36, 190–197. 10.3109/02713683.2010.544442 21275516

[B13] Castro-Luna G. Jimenez-Rodriguez D. Castano-Fernandez A. B. Perez-Rueda A. (2021). Diagnosis of subclinical keratoconus based on machine learning techniques. J. Clin. Med. 10, 4281. 10.3390/jcm10184281 34575391 PMC8468312

[B14] Chan E. Chong E. W. Lingham G. Stevenson L. J. Sanfilippo P. G. Hewitt A. W. (2021). Prevalence of keratoconus based on scheimpflug imaging: The raine study. Ophthalmology 128, 515–521. 10.1016/j.ophtha.2020.08.020 32860813

[B15] Chase C. Elsawy A. Eleiwa T. Ozcan E. Tolba M. Abou Shousha M. (2021). Comparison of autonomous AS-OCT deep learning algorithm and clinical dry eye tests in diagnosis of dry eye disease. Clin. Ophthalmol. 15, 4281–4289. 10.2147/OPTH.S321764 34707347 PMC8545140

[B16] Chatzis N. Hafezi F. (2012). Progression of keratoconus and efficacy of pediatric [corrected] corneal collagen cross-linking in children and adolescents. J. Refract. Surg. 28, 753–758. 10.3928/1081597X-20121011-01 23347367

[B17] Chen X. Zhao J. Iselin K. C. Borroni D. Romano D. Gokul A. (2021). Keratoconus detection of changes using deep learning of colour-coded maps. BMJ Open Ophthalmol. 6, e000824. 10.1136/bmjophth-2021-000824 PMC8278890 34337155

[B18] Chidambaram J. D. Venkatesh Prajna N. Srikanthi P. Lanjewar S. Shah M. Elakkiya S. (2018). Epidemiology, risk factors, and clinical outcomes in severe microbial keratitis in South India. Ophthalmic Epidemiol. 25, 297–305. 10.1080/09286586.2018.1454964 29580152 PMC5985925

[B19] Christopher M. Belghith A. Bowd C. Proudfoot J. A. Goldbaum M. H. Weinreb R. N. (2018). Performance of deep learning architectures and transfer learning for detecting glaucomatous optic neuropathy in fundus photographs. Sci. Rep. 8, 16685. 10.1038/s41598-018-35044-9 30420630 PMC6232132

[B20] Coroneo M. (2011). Ultraviolet radiation and the anterior eye. Eye Contact Lens 37, 214–224. 10.1097/ICL.0b013e318223394e 21670690

[B21] Craig J. P. Nelson J. D. Azar D. T. Belmonte C. Bron A. J. Chauhan S. K. (2017a). TFOS DEWS II report executive summary. Ocul. Surf. 15, 802–812. 10.1016/j.jtos.2017.08.003 28797892

[B22] Craig J. P. Nichols K. K. Akpek E. K. Caffery B. Dua H. S. Joo C. K. (2017b). TFOS DEWS II definition and classification report. Ocul. Surf. 15, 276–283. 10.1016/j.jtos.2017.05.008 28736335

[B23] Da Cruz L. B. Souza J. C. DE Paiva A. C. DE Almeida J. D. S. Junior G. B. Aires K. R. T. (2020a). Tear film classification in interferometry eye images using phylogenetic diversity indexes and ripley's K function. IEEE J. Biomed. Health Inf. 24, 3491–3498. 10.1109/JBHI.2020.3026940 32976110

[B24] Da Cruz L. B. Souza J. C. DE Sousa J. A. Santos A. M. DE Paiva A. C. DE Almeida J. D. S. (2020b). Interferometer eye image classification for dry eye categorization using phylogenetic diversity indexes for texture analysis. Comput. Methods Programs Biomed. 188, 105269. 10.1016/j.cmpb.2019.105269 31846832

[B25] DE Sanctis U. Loiacono C. Richiardi L. Turco D. Mutani B. Grignolo F. M. (2008). Sensitivity and specificity of posterior corneal elevation measured by Pentacam in discriminating keratoconus/subclinical keratoconus. Ophthalmology 115, 1534–1539. 10.1016/j.ophtha.2008.02.020 18405974

[B26] Deangelis K. D. Rider A. Potter W. Jensen J. Fowler B. T. Fleming J. C. (2019). Eyelid spontaneous blink analysis and age-related changes through high-speed imaging. Ophthalmic Plast. Reconstr. Surg. 35, 487–490. 10.1097/IOP.0000000000001349 30844914

[B27] Delic N. C. Lyons J. G. DI Girolamo N. Halliday G. M. (2017). Damaging effects of ultraviolet radiation on the cornea. Photochem. Photobiol. 93, 920–929. 10.1111/php.12686 27935054

[B28] Dong L. He W. Zhang R. Ge Z. Wang Y. X. Zhou J. (2022). Artificial intelligence for screening of multiple retinal and optic nerve diseases. JAMA Netw. Open 5, e229960. 10.1001/jamanetworkopen.2022.9960 35503220 PMC9066285

[B29] Dos Santos V. A. Schmetterer L. Stegmann H. Pfister M. Messner A. Schmidinger G. (2019). CorneaNet: Fast segmentation of cornea OCT scans of healthy and keratoconic eyes using deep learning. Biomed. Opt. Express 10, 622–641. 10.1364/BOE.10.000622 30800504 PMC6377876

[B30] Fang X. Deshmukh M. Chee M. L. Soh Z. D. Teo Z. L. Thakur S. (2021). Deep learning algorithms for automatic detection of pterygium using anterior segment photographs from slit-lamp and hand-held cameras. Br. J. Ophthalmol. 106, 1642–1647. 10.1136/bjophthalmol-2021-318866 34244208 PMC9685734

[B31] Ferdi A. C. Nguyen V. Gore D. M. Allan B. D. Rozema J. J. Watson S. L. (2019). Keratoconus natural progression: A systematic review and meta-analysis of 11 529 eyes. Ophthalmology 126, 935–945. 10.1016/j.ophtha.2019.02.029 30858022

[B32] Flaxman S. R. Bourne R. R. A. Resnikoff S. Ackland P. Braithwaite T. Cicinelli M. V. VISION LOSS EXPERT GROUP OF THE GLOBAL BURDEN OF DISEASE, S (2017). Global causes of blindness and distance vision impairment 1990-2020: A systematic review and meta-analysis. Lancet. Glob. Health 5, e1221–e1234. 10.1016/S2214-109X(17)30393-5 29032195

[B33] Ghosh A. K. Thammasudjarit R. Jongkhajornpong P. Attia J. Thakkinstian A. (2022). Deep learning for discrimination between fungal keratitis and bacterial keratitis: DeepKeratitis. Cornea 41, 616–622. 10.1097/ICO.0000000000002830 34581296 PMC8969839

[B34] Goh Y. W. Gokul A. Yadegarfar M. E. Vellara H. Shew W. Patel D. (2020). Prospective clinical study of keratoconus progression in patients awaiting corneal cross-linking. Cornea 39, 1256–1260. 10.1097/ICO.0000000000002376 32482959

[B35] Gordon-Shaag A. Millodot M. Ifrah R. Shneor E. (2012). Aberrations and topography in normal, keratoconus-suspect, and keratoconic eyes. Optom. Vis. Sci. 89, 411–418. 10.1097/OPX.0b013e318249d727 22311193

[B36] Graue-Hernandez E. O. Cordoba A. Jimenez-Corona A. Ramirez-Miranda A. Navas A. Serna-Ojeda J. C. (2019). Practice patterns in the management of primary pterygium: A survey study. Cornea 38, 1339–1344. 10.1097/ICO.0000000000002091 31403528

[B37] Gu H. Guo Y. Gu L. Wei A. Xie S. Ye Z. (2020). Deep learning for identifying corneal diseases from ocular surface slit-lamp photographs. Sci. Rep. 10, 17851. 10.1038/s41598-020-75027-3 33082530 PMC7576153

[B38] Hashemi H. Heydarian S. Hooshmand E. Saatchi M. Yekta A. Aghamirsalim M. (2020). The prevalence and risk factors for keratoconus: A systematic review and meta-analysis. Cornea 39, 263–270. 10.1097/ICO.0000000000002150 31498247

[B39] Herber R. Pillunat L. E. Raiskup F. (2021). Development of a classification system based on corneal biomechanical properties using artificial intelligence predicting keratoconus severity. Eye Vis. 8, 21. 10.1186/s40662-021-00244-4 PMC8167942 34059127

[B40] Hirst L. W. (2003). The treatment of pterygium. Surv. Ophthalmol. 48, 145–180. 10.1016/s0039-6257(02)00463-0 12686302

[B41] Hood D. C. DE Moraes C. G. (2018). Efficacy of a deep learning system for detecting glaucomatous optic neuropathy based on color fundus photographs. Ophthalmology 125, 1207–1208. 10.1016/j.ophtha.2018.04.020 30032794

[B42] Hu J. W. Zhu X. P. Pan S. Y. Yang H. Xiao X. H. (2021). Prevalence and risk factors of dry eye disease in young and middle-aged office employee: A xi'an study. Int. J. Ophthalmol. 14, 567–573. 10.18240/ijo.2021.04.14 33875949 PMC8025180

[B43] Huang Y. He H. Sheha H. Tseng S. C. (2013). Ocular demodicosis as a risk factor of pterygium recurrence. Ophthalmology 120, 1341–1347. 10.1016/j.ophtha.2013.01.001 23664471

[B44] Hung K. H. Lin C. Roan J. Kuo C. F. Hsiao C. H. Tan H. Y. (2022). Application of a deep learning system in pterygium grading and further prediction of recurrence with slit lamp photographs. Diagn. (Basel) 12, 888. 10.3390/diagnostics12040888 PMC9029774 35453936

[B45] Hung N. Shih A. K. Lin C. Kuo M. T. Hwang Y. S. Wu W. C. (2021). Using Slit-Lamp Images for Deep Learning-Based Identification of Bacterial and Fungal Keratitis: Model Development and Validation with Different Convolutional Neural Networks, 11.Diagn. (Basel). 10.3390/diagnostics11071246 PMC8307675 34359329

[B46] Jais F. N. Che Azemin M. Z. Hilmi M. R. Mohd Tamrin M. I. Kamal K. M. (2021). Postsurgery classification of best-corrected visual acuity changes based on pterygium characteristics using the machine learning technique. ScientificWorldJournal. 2021, 6211006. 10.1155/2021/6211006 34819813 PMC8608506

[B47] Kamiya K. Ayatsuka Y. Kato Y. Shoji N. Miyai T. Ishii H. (2021). Prediction of keratoconus progression using deep learning of anterior segment optical coherence tomography maps. Ann. Transl. Med. 9, 1287. 10.21037/atm-21-1772 34532424 PMC8422102

[B48] Kamiya K. Ayatsuka Y. Kato Y. Fujimura F. Takahashi M. Shoji N. (2019). Keratoconus detection using deep learning of colour-coded maps with anterior segment optical coherence tomography: A diagnostic accuracy study. BMJ Open 9, e031313. 10.1136/bmjopen-2019-031313 PMC6773416 31562158

[B49] Kampitak K. Leelawongtawun W. Leeamornsiri S. Suphachearaphan W. Thitiwichienlert S. (2016). A comparative study of higher order aberrations between pterygium and non-pterygium eyes. J. Med. Assoc. Thai 99 (4), S178–S181. 29926699

[B50] Kang L. Ballouz D. Woodward M. A. (2022). Artificial intelligence and corneal diseases. Curr. Opin. Ophthalmol. 33, 407–417. 10.1097/ICU.0000000000000885 35819899 PMC9357186

[B51] Kato N. Masumoto H. Tanabe M. Sakai C. Negishi K. Torii H. (2021). Predicting keratoconus progression and need for corneal crosslinking using deep learning. J. Clin. Med. 10, 844. 10.3390/jcm10040844 33670732 PMC7923054

[B52] Keel S. Lee P. Y. Scheetz J. Li Z. Kotowicz M. A. Macisaac R. J. (2018). Feasibility and patient acceptability of a novel artificial intelligence-based screening model for diabetic retinopathy at endocrinology outpatient services: A pilot study. Sci. Rep. 8, 4330. 10.1038/s41598-018-22612-2 29531299 PMC5847544

[B53] Khor W. B. Prajna V. N. Garg P. Mehta J. S. Xie L. Liu Z. (2018). The asia cornea society infectious keratitis study: A prospective multicenter study of infectious keratitis in asia. Am. J. Ophthalmol. 195, 161–170. 10.1016/j.ajo.2018.07.040 30098351

[B54] Koprowski R. Wilczynski S. Olczyk P. Nowinska A. Weglarz B. Wylegala E. (2016). A quantitative method for assessing the quality of meibomian glands. Comput. Biol. Med. 75, 130–138. 10.1016/j.compbiomed.2016.06.001 27286185

[B55] Kuo B. I. Chang W. Y. Liao T. S. Liu F. Y. Liu H. Y. Chu H. S. (2020a). Keratoconus screening based on deep learning approach of corneal topography. Transl. Vis. Sci. Technol. 9, 53. 10.1167/tvst.9.2.53 33062398 PMC7533740

[B56] Kuo M. T. Hsu B. W. Lin Y. S. Fang P. C. Yu H. J. Chen A. (2021). Comparisons of deep learning algorithms for diagnosing bacterial keratitis via external eye photographs. Sci. Rep. 11, 24227. 10.1038/s41598-021-03572-6 34930952 PMC8688438

[B57] Kuo M. T. Hsu B. W. Yin Y. K. Fang P. C. Lai H. Y. Chen A. (2020b). A deep learning approach in diagnosing fungal keratitis based on corneal photographs. Sci. Rep. 10, 14424. 10.1038/s41598-020-71425-9 32879364 PMC7468230

[B58] Lavric A. Valentin P. (2019). KeratoDetect: Keratoconus detection algorithm using convolutional neural networks. Comput. Intell. Neurosci. 2019, 8162567. 10.1155/2019/8162567 30809255 PMC6364125

[B59] Li Z. Jiang J. Chen K. Chen Q. Zheng Q. Liu X. (2021). Preventing corneal blindness caused by keratitis using artificial intelligence. Nat. Commun. 12, 3738. 10.1038/s41467-021-24116-6 34145294 PMC8213803

[B60] Liu Z. Cao Y. Li Y. Xiao X. Qiu Q. Yang M. (2020). Automatic diagnosis of fungal keratitis using data augmentation and image fusion with deep convolutional neural network. Comput. Methods Programs Biomed. 187, 105019. 10.1016/j.cmpb.2019.105019 31421868

[B61] Lv J. Zhang K. Chen Q. Chen Q. Huang W. Cui L. (2020). Deep learning-based automated diagnosis of fungal keratitis with *in vivo* confocal microscopy images. Ann. Transl. Med. 8, 706. 10.21037/atm.2020.03.134 32617326 PMC7327373

[B62] Mahar P. S. Manzar N. (2013). Pterygium recurrence related to its size and corneal involvement. J. Coll. Physicians Surg. Pak. 23, 120–123. 10.2.2013/JCPSP.120123 23374515

[B63] Martins T. Schor P. Mendes L. G. A. Fowler S. Silva R. (2022). Use of artificial intelligence in ophthalmology: A narrative review. Sao Paulo Med. J. 140, 837–845. 10.1590/1516-3180.2021.0713.R1.22022022 36043665 PMC9671570

[B64] Maruoka S. Tabuchi H. Nagasato D. Masumoto H. Chikama T. Kawai A. (2020). Deep neural network-based method for detecting obstructive meibomian gland dysfunction with *in vivo* laser confocal microscopy. Cornea 39, 720–725. 10.1097/ICO.0000000000002279 32040007

[B65] Medeiros F. A. Jammal A. A. Mariottoni E. B. (2021). Detection of progressive glaucomatous optic nerve damage on fundus photographs with deep learning. Ophthalmology 128, 383–392. 10.1016/j.ophtha.2020.07.045 32735906 PMC7386268

[B66] Mohammadpour M. Heidari Z. Hashemi H. (2018). Updates on managements for keratoconus. J. Curr. Ophthalmol. 30, 110–124. 10.1016/j.joco.2017.11.002 29988906 PMC6034171

[B67] Mohd Radzi H. Khairidzan M. K. Mohd Zulfaezal C. A. Azrin E. A. (2019). Corneo-pterygium total area measurements utilising image analysis method. J. Optom. 12, 272–277. 10.1016/j.optom.2019.04.001 31097348 PMC6978598

[B68] Nagasato D. Tabuchi H. Ohsugi H. Masumoto H. Enno H. Ishitobi N. (2018). Deep neural network-based method for detecting central retinal vein occlusion using ultrawide-field fundus ophthalmoscopy. J. Ophthalmol. 2018, 1875431. 10.1155/2018/1875431 30515316 PMC6236766

[B69] Nagasato D. Tabuchi H. Ohsugi H. Masumoto H. Enno H. Ishitobi N. (2019). Deep-learning classifier with ultrawide-field fundus ophthalmoscopy for detecting branch retinal vein occlusion. Int. J. Ophthalmol. 12, 94–99. 10.18240/ijo.2019.01.15 30662847 PMC6326931

[B70] Nichols K. K. Foulks G. N. Bron A. J. Glasgow B. J. Dogru M. Tsubota K. (2011). The international workshop on meibomian gland dysfunction: Executive summary. Invest. Ophthalmol. Vis. Sci. 52, 1922–1929. 10.1167/iovs.10-6997a 21450913 PMC3072157

[B71] Nichols K. K. Mitchell G. L. Zadnik K. (2004). The repeatability of clinical measurements of dry eye. Cornea 23, 272–285. 10.1097/00003226-200404000-00010 15084861

[B72] Papali'I-Curtin A. T. Cox R. Ma T. Woods L. Covello A. Hall R. C. (2019). Keratoconus prevalence among high School students in New Zealand. Cornea 38, 1382–1389. 10.1097/ICO.0000000000002054 31335534

[B73] Pascolini D. Mariotti S. P. (2012). Global estimates of visual impairment: 2010. Br. J. Ophthalmol. 96, 614–618. 10.1136/bjophthalmol-2011-300539 22133988

[B74] Pinero D. P. Nieto J. C. Lopez-Miguel A. (2012). Characterization of corneal structure in keratoconus. J. Cataract. Refract. Surg. 38, 2167–2183. 10.1016/j.jcrs.2012.10.022 23195256

[B75] Pur D. R. Krance S. H. Pucchio A. Miranda R. N. Felfeli T. (2022). Current uses of artificial intelligence in the analysis of biofluid markers involved in corneal and ocular surface diseases: A systematic review. Eye (Lond). 10.1038/s41433-022-02307-9 PMC10333344 36380089

[B76] Raman R. Srinivasan S. Virmani S. Sivaprasad S. Rao C. Rajalakshmi R. (2019). Fundus photograph-based deep learning algorithms in detecting diabetic retinopathy. Eye (Lond) 33, 97–109. 10.1038/s41433-018-0269-y 30401899 PMC6328553

[B77] Redd T. K. Campbell J. P. Brown J. M. Kim S. J. Ostmo S. Chan R. V. P. (2018). IMAGING & INFORMATICS IN RETINOPATHY OF PREMATURITY RESEARCH, CEvaluation of a deep learning image assessment system for detecting severe retinopathy of prematurity. Br. J. Ophthalmol. 103, 580–584. 10.1136/bjophthalmol-2018-313156 PMC7880608 30470715

[B78] Resnikoff S. Lansingh V. C. Washburn L. Felch W. Gauthier T. M. Taylor H. R. (2020). Estimated number of ophthalmologists worldwide (international council of ophthalmology update): Will we meet the needs? Br. J. Ophthalmol. 104, 588–592. 10.1136/bjophthalmol-2019-314336 31266774 PMC7147181

[B79] Rezvan F. Khabazkhoob M. Hooshmand E. Yekta A. Saatchi M. Hashemi H. (2018). Prevalence and risk factors of pterygium: A systematic review and meta-analysis. Surv. Ophthalmol. 63, 719–735. 10.1016/j.survophthal.2018.03.001 29551597

[B80] Rocha-DE-Lossada C. Prieto-Godoy M. Sanchez-Gonzalez J. M. Romano V. Borroni D. Rachwani-Anil R. (2021). Tomographic and aberrometric assessment of first-time diagnosed paediatric keratoconus based on age ranges: A multicentre study. Acta Ophthalmol. 99, e929–e936. 10.1111/aos.14715 33377591

[B81] Rodriguez J. D. Lane K. J. Ousler G. W., 3R. D. Angjeli E. Smith L. M. Abelson M. B. (2018). Blink: Characteristics, controls, and relation to dry eyes. Curr. Eye Res. 43, 52–66. 10.1080/02713683.2017.1381270 29043838

[B82] Safi H. Kheirkhah A. Mahbod M. Molaei S. Hashemi H. Jabbarvand M. (2016). Correlations between histopathologic changes and clinical features in pterygia. J. Ophthalmic Vis. Res. 11, 153–158. 10.4103/2008-322X.183917 27413494 PMC4926561

[B83] Seet L. F. Tong L. Su R. Wong T. T. (2012). Involvement of SPARC and MMP-3 in the pathogenesis of human pterygium. Invest. Ophthalmol. Vis. Sci. 53, 587–595. 10.1167/iovs.11-7941 22222271

[B84] Setu M. A. K. Horstmann J. Schmidt S. Stern M. E. Steven P. (2021). Deep learning-based automatic meibomian gland segmentation and morphology assessment in infrared meibography. Sci. Rep. 11, 7649. 10.1038/s41598-021-87314-8 33828177 PMC8027879

[B85] Sheng B. Chen X. Li T. Ma T. Yang Y. Bi L. (2022). An overview of artificial intelligence in diabetic retinopathy and other ocular diseases. Front. Public Health 10, 971943. 10.3389/fpubh.2022.971943 36388304 PMC9650481

[B86] Sjo N. C. VON Buchwald C. Prause J. U. Norrild B. Vinding T. Heegaard S. (2007). Human papillomavirus and pterygium. Is the virus a risk factor? Br. J. Ophthalmol. 91, 1016–1018. 10.1136/bjo.2006.108829 17179167 PMC1954836

[B87] Stapleton F. Alves M. Bunya V. Y. Jalbert I. Lekhanont K. Malet F. (2017). TFOS DEWS II epidemiology report. Ocul. Surf. 15, 334–365. 10.1016/j.jtos.2017.05.003 28736337

[B88] Sullivan B. D. Whitmer D. Nichols K. K. Tomlinson A. Foulks G. N. Geerling G. (2010). An objective approach to dry eye disease severity. Invest. Ophthalmol. Vis. Sci. 51, 6125–6130. 10.1167/iovs.10-5390 20631232

[B89] Tahvildari M. Singh R. B. Saeed H. N. (2021). Application of artificial intelligence in the diagnosis and management of corneal diseases. Semin. Ophthalmol. 36, 641–648. 10.1080/08820538.2021.1893763 33689543

[B90] Tan Z. Chen X. Li K. Liu Y. Cao H. Li J. (2022). Artificial intelligence-based diagnostic model for detecting keratoconus using videos of corneal force deformation. Transl. Vis. Sci. Technol. 11, 32. 10.1167/tvst.11.9.32 PMC9527334 36178782

[B91] Tena D. Rodriguez N. Toribio L. Gonzalez-Praetorius A. (2019). Infectious keratitis: Microbiological review of 297 cases. Jpn. J. Infect. Dis. 72, 121–123. 10.7883/yoken.JJID.2018.269 30381686

[B92] Tepelus T. C. Chiu G. B. Huang J. Huang P. Sadda S. R. Irvine J. (2017). Correlation between corneal innervation and inflammation evaluated with confocal microscopy and symptomatology in patients with dry eye syndromes: A preliminary study. Graefes Arch. Clin. Exp. Ophthalmol. 255, 1771–1778. 10.1007/s00417-017-3680-3 28528377

[B93] Ting D. S. J. Foo V. H. Yang L. W. Y. Sia J. T. Ang M. Lin H. (2021). Artificial intelligence for anterior segment diseases: Emerging applications in ophthalmology. Br. J. Ophthalmol. 105, 158–168. 10.1136/bjophthalmol-2019-315651 32532762

[B94] Ting D. S. J. Settle C. Morgan S. J. Baylis O. Ghosh S. (2018). A 10-year analysis of microbiological profiles of microbial keratitis: The north east england study. Eye (Lond) 32, 1416–1417. 10.1038/s41433-018-0085-4 29610521 PMC6085375

[B95] Tiwari M. Piech C. Baitemirova M. Prajna N. V. Srinivasan M. Lalitha P. (2022). Differentiation of active corneal infections from healed scars using deep learning. Ophthalmology 129, 139–146. 10.1016/j.ophtha.2021.07.033 34352302 PMC8792172

[B96] Vehof J. Utheim T. P. Bootsma H. Hammond C. J. (2020). Advances, limitations and future perspectives in the diagnosis and management of dry eye in Sjogren's syndrome. Clin. Exp. Rheumatol. 38 (126), 301–309. 33025899

[B97] Wan C. Shao Y. Wang C. Jing J. Yang W. (2022). A novel system for measuring pterygium's progress using deep learning. Front. Med. 9, 819971. 10.3389/fmed.2022.819971 PMC8882585 35237630

[B98] Wan Zaki W. M. D. Mat Daud M. Abdani S. R. Hussain A. Mutalib H. A. (2018). Automated pterygium detection method of anterior segment photographed images. Comput. Methods Programs Biomed. 154, 71–78. 10.1016/j.cmpb.2017.10.026 29249348

[B99] Wang J. Ji J. Zhang M. Lin J. W. Zhang G. Gong W. (2021a). Automated explainable multidimensional deep learning platform of retinal images for retinopathy of prematurity screening. JAMA Netw. Open 4, e218758. 10.1001/jamanetworkopen.2021.8758 33950206 PMC8100867

[B100] Wang J. Yeh T. N. Chakraborty R. Yu S. X. Lin M. C. (2019). A deep learning approach for meibomian gland atrophy evaluation in meibography images. Transl. Vis. Sci. Technol. 8, 37. 10.1167/tvst.8.6.37 PMC6922272 31867138

[B101] Wang Y. C. Zhao F. K. Liu Q. Yu Z. Y. Wang J. Zhang J. S. (2021b). Bibliometric analysis and mapping knowledge domain of pterygium: 2000-2019. Int. J. Ophthalmol. 14, 903–914. 10.18240/ijo.2021.06.17 34150547 PMC8165632

[B102] Xu F. Qin Y. He W. Huang G. Lv J. Xie X. (2021a). A deep transfer learning framework for the automated assessment of corneal inflammation on *in vivo* confocal microscopy images. PLoS One 16, e0252653. 10.1371/journal.pone.0252653 34081736 PMC8174724

[B103] Xu W. Jin L. Zhu P. Z. He K. Yang W. H. Wu M. N. (2021b). Implementation and application of an intelligent pterygium diagnosis system based on deep learning. Front. Psychol. 12, 759229. 10.3389/fpsyg.2021.759229 34744935 PMC8569253

[B104] Xu W. Yan Z. Chen N. Luo Y. Ji Y. Wang M. (2022). Development and application of an intelligent diagnosis system for retinal vein occlusion based on deep learning. Dis. Markers 2022, 4988256. 10.1155/2022/4988256 36061353 PMC9433258

[B105] Yan Q. Weeks D. E. Xin H. Swaroop A. Chew E. Y. Huang H. (2020). Deep-learning-based prediction of late age-related macular degeneration progression. Nat. Mach. Intell. 2, 141–150. 10.1038/s42256-020-0154-9 32285025 PMC7153739

[B106] Yim J. Chopra R. Spitz T. Winkens J. Obika A. Kelly C. (2020). Predicting conversion to wet age-related macular degeneration using deep learning. Nat. Med. 26, 892–899. 10.1038/s41591-020-0867-7 32424211

[B107] Yousefi S. Yousefi E. Takahashi H. Hayashi T. Tampo H. Inoda S. (2018). Keratoconus severity identification using unsupervised machine learning. PLoS One 13, e0205998. 10.1371/journal.pone.0205998 30399144 PMC6219768

[B108] Yue X. L. Gao Z. Q. (2019). Identification of pathogenic genes of pterygium based on the Gene Expression Omnibus database. Int. J. Ophthalmol. 12, 529–535. 10.18240/ijo.2019.04.01 31024802 PMC6469547

[B109] Zeev M. S. Miller D. D. Latkany R. (2014). Diagnosis of dry eye disease and emerging technologies. Clin. Ophthalmol. 8, 581–590. 10.2147/OPTH.S45444 24672224 PMC3964175

[B110] Zhang Z. Z. Kuang R. F. Wei Z. Y. Wang L. Y. Su G. Y. Ou Z. H. (2022). Detection of the spontaneous blinking pattern of dry eye patients using the machine learning method. Zhonghua. Yan Ke Za Zhi. 58, 120–129. 10.3760/cma.j.cn112142-20211110-00537 35144352

[B111] Zheng B. Liu Y. He K. Wu M. Jin L. Jiang Q. (2021). Research on an intelligent lightweight-assisted pterygium diagnosis model based on anterior segment images. Dis. Markers 2021, 7651462. 10.1155/2021/7651462 34367378 PMC8342163

